# Accelerated exploration of multi-principal element alloys with solid solution phases

**DOI:** 10.1038/ncomms7529

**Published:** 2015-03-05

**Authors:** O.N. Senkov, J.D. Miller, D.B. Miracle, C. Woodward

**Affiliations:** 1 Air Force Research Laboratory, Materials and Manufacturing Directorate, 2230 Tenth Street, Wright-Patterson AFB, Ohio 45433, USA

## Abstract

Recent multi-principal element, high entropy alloy (HEA) development strategies vastly expand the number of candidate alloy systems, but also pose a new challenge—how to rapidly screen thousands of candidate alloy systems for targeted properties. Here we develop a new approach to rapidly assess structural metals by combining calculated phase diagrams with simple rules based on the phases present, their transformation temperatures and useful microstructures. We evaluate over 130,000 alloy systems, identifying promising compositions for more time-intensive experimental studies. We find the surprising result that solid solution alloys become less likely as the number of alloy elements increases. This contradicts the major premise of HEAs—that increased configurational entropy increases the stability of disordered solid solution phases. As the number of elements increases, the configurational entropy rises slowly while the probability of at least one pair of elements favouring formation of intermetallic compounds increases more rapidly, explaining this apparent contradiction.

## Abstract (web summary)

In high entropy alloys a mix of a large number of five or more principal atomic elements is used to tune the properties. Here, the authors present a solution to the problem of predicting the properties of the huge number of potential alloys by developing an efficient screening approach based on automated calculations.

## Graphical abstract

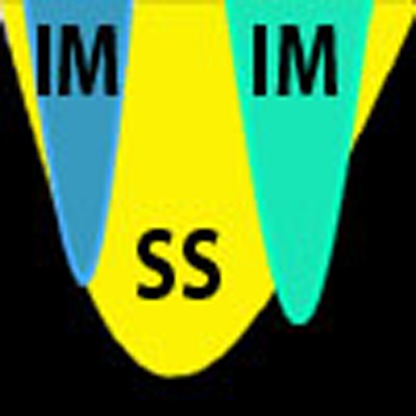

Conventional alloys have one principal element, with minor modifications achieved by adding relatively small amounts of other elements. As many as a dozen other elements may be added, but conventional alloys still usually have a majority atom fraction of the base element. This strategy optimizes a suite of properties while retaining the characteristic properties of the base element that make this alloy family attractive. Multi-principle element alloys (MPEAs, among which alloys with ≥5 elements are also called high entropy alloys, HEAs) are a new alloy development philosophy^1^,^2^, where the base alloy has significant atom fractions of several elements. Four or more base elements are commonly used, and although MPEAs often have equal concentrations of *N* base elements, this is not required. A common rationale for increasing *N* is to maximize the configurational entropy in order to improve the stability of disordered solid solution (SS) phases, thus suppressing formation of intermetallic (IM) phases.

This new alloying strategy vastly increases the number of possible alloy systems, giving a rich composition and phase space that has not yet been explored. For example, a palette of 12 elements gives 12 conventional alloy systems where 1 element dominates. Significant changes in the phases present and their reaction temperatures of MPEAs give a unique alloy system for each combination of elements. If an alloy base is made by combining 3 of the 12 elements in the palette above, then there are 220 distinct combinations of elements (that is, the binomial coefficient, see Methods). Each of these 220 alloy families can be modified by altering the relative concentrations of the 3 base elements or by adding relatively minor amounts of other elements as is done in conventional alloys. The same palette of 12 elements gives 495 alloy families with 4 base elements; 792 systems with 5 base elements, 924 6-base-element systems and a total of 4,017 alloy systems consisting of between 3 and 12 base elements. Thus, while the number of conventional alloy systems equals the number of elements in a palette, the number of MPEA systems is a function of *N*! vastly increasing the number of systems.

This greatly expands opportunities for discovering alloys with new and useful properties, but introduces a new challenge. It is relatively simple to choose an alloy system for an intended application when it is based on a single element—the properties of the base element are a useful guide for achieving target properties. But this is not so simple for MPEAs. Some properties such as density, elastic modulus and cost can be reasonably estimated from the rule-of-mixtures of the base elements, however, this is not the case for important properties such as strength, ductility and maximum use temperature. Even simple experimental evaluations of these properties are time-intensive, requiring weeks or months to produce and characterize a small number of candidate alloys. Evaluating hundreds of thousands of candidates is a truly daunting task, requiring completely new approaches.

Combinatorial methods are common in many fields^3^,^4^,^5^,^6^,^7^. These ‘combi’ approaches produce thousands to millions of compositions in a ‘materials library’—a single sample with controlled composition gradients. Specialized experimental techniques measure properties at local compositions of the materials library through miniaturization and automation, dramatically accelerating characterization. However, structural properties depend sensitively on microstructure, and microstructural length scales (grain size, sub-grain size, precipitate size and spacing and so on.) set limits on miniaturization. For example, average properties are measured when a test sample is much larger than the grain size; single crystal properties are measured when a sample is much smaller than the grain size; and strong statistical variations in properties are typical when sample dimensions are of the same order of magnitude as the grain size. These intrinsic microstructural length scales block the full potential of combinatorial methods for assessing structural properties.

Here we establish a fundamentally new combinatorial approach to rapidly screen large number of candidate structural metal alloys using the calculated phase diagram method (CALPHAD, see Methods) to calculate the phase diagram of each alloy. This gives a rudimentary microstructural assessment, since every calculated phase diagram gives the phases present and their reaction temperatures. Simple rules evaluate an alloy’s potential for structural applications. For example, the solidus temperature (*T*_m_) must be above the maximum use temperature (*T*_use_) and there should be no first-order phase transformations below *T*_use_ to avoid property changes during service. Good ductility and toughness are often associated with a SS primary phase, and the most potent strengthening mechanisms require the controlled distribution of a second phase. Typically, structural alloys are designed such that *T*_use_<*T*_s_<*T*_m_, where *T*_s_ is the solvus temperature of the strengthening phase. The distribution and volume fraction of strengthening phases can be controlled by heat treatments above *T*_s_, to dissolve the strengthening phase, followed by quenching and annealing between *T*_use_ and *T*_s_ to nucleate and grow the strengthening phase. Rules can also be applied regarding the number and types of phases and practical metrics such as alloy density, elastic properties and alloy cost, which can be estimated by the rule of mixtures of the elements present. Details of the specific rules, the CALPHAD calculations and approaches to establish the credibility of the CALPHAD calculations are provided in the Methods section. The concepts used here have been proposed earlier^8^, the present work is the first application of these concepts.

## Results

### Elemental basis and CALPHAD analysis

We build a palette of elements to develop intermediate- and high-temperature structural metals (see Supplementary Table 1). The concepts used to build this palette are taken from ref. 8 and are guided by the elements available in thermodynamic databases. Al and Si are included since they often form compounds that satisfy melting temperature (*T*_m_) and elastic modulus criteria. We calculate phase diagrams for every equimolar combination of 3, 4, 5 or 6 elements from this palette, giving over 130,000 alloy systems. Each alloy system has a vast number of distinct alloy compositions. For simplicity, we demonstrate the present approach by calculating the phase diagram for only the equimolar composition in each alloy system. We develop credibility criteria (see Methods) to improve confidence in the CALPHAD calculations, which include the fraction of assessed binary systems (*f*_AB_) included in the database for the specific alloy under consideration. To improve confidence in the calculated phase diagrams, we only consider results in the present work where all the binary systems represented by the alloy under consideration have complete description in the thermodynamic database from which the phase diagram was calculated. Thus, *f*_AB_=1 for every alloy in the present work. We characterize each equimolar alloy that passes this test by the number and by the types of phases present (SS, IM or SS+IM) at *T*_m_ and at 600 °C. To explore ideas common in the HEA literature, we place a special emphasis on alloys that contain only SS phases. Finally, we demonstrate the ability to identify promising alloys by reporting which of the 130,000 calculated alloys satisfy criteria for high-temperature structural metals. Where available, we compare CALPHAD calculations with experimental results. A total of 134,547 phase diagram calculations were performed using the CALPHAD approach in the present work (see Methods). Only 5,540 CALPHAD calculations pass the *f*_AB_=1 filter, and only 4,983 of these calculations are for unique, equimolar alloys since the same alloy can be calculated from more than 1 thermodynamic database (see Supplementary Table 2).

### SS alloys become less common as *N* increases

**Figure 1:**
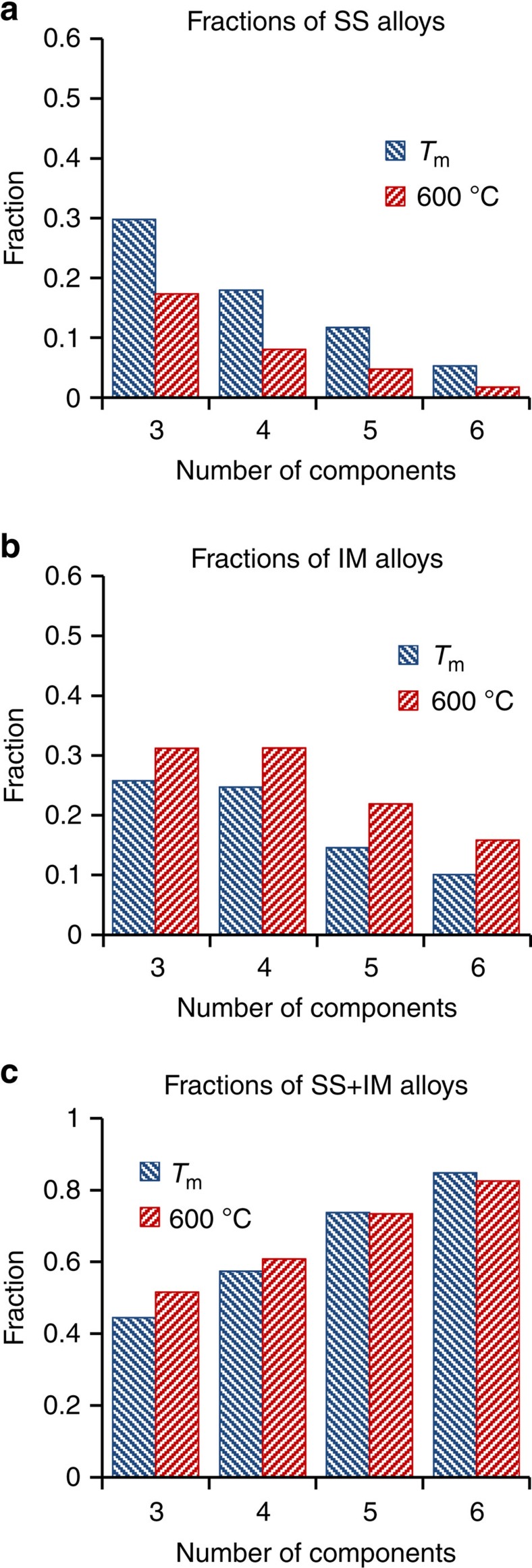
Distributions of multi-principal element alloys by categories. Fractions of (**a**) SS, (**b**) IM and (**c**) (SS+IM) equimolar alloys in 3- to 6-component alloy systems at *T*_m_ and 600 °C.

All the processed alloys can be divided into three categories: (i) SS alloys, which consist only of one or more SS phases; (ii) IM alloys, consisting of only one or more IM phases (including IM phases that are stable over a range of compositions and thus have some ability to produce ordered SS, see Methods); and (iii) SS+IM alloys, which contain both SS and IM phases. The number of 3- to 6-component SS and IM alloys are given in Table 1 and the fractions of the SS, IM and (SS+IM) alloy categories at *T*_m_ and 600 °C are given in Figs 1a–c, respectively. At *T*_m_, 30% of 3-component alloys are SS alloys, 26% are IM alloys and 44% are (SS+IM) alloys. The fractions of both SS and IM alloys decrease while the fraction of (SS+IM) alloys increases with an increase in the number of alloying elements, *N*, and among the 6-component alloys only 5% are SS and 10% are IM. With a decrease in temperature to 600 °C, the number of SS alloys considerably decreases, while the number of IM alloys increases and the number of (SS+IM) alloys remains almost unchanging. The dependences of the fraction of these alloy categories on the number of components at 600 °C and *T*_m_ are similar (see Fig. 1). Both the number of SS alloys (Table 1) and the fraction of SS alloys (Fig. 1a) decrease with increasing *N*, contradicting a major concept of the HEA approach—that increasing the number of constituents increases configurational entropy and stabilizes SS phases relative to ordered IM phases^1^,^2^.

### SS alloys usually have one to three phases

**Figure 2:**
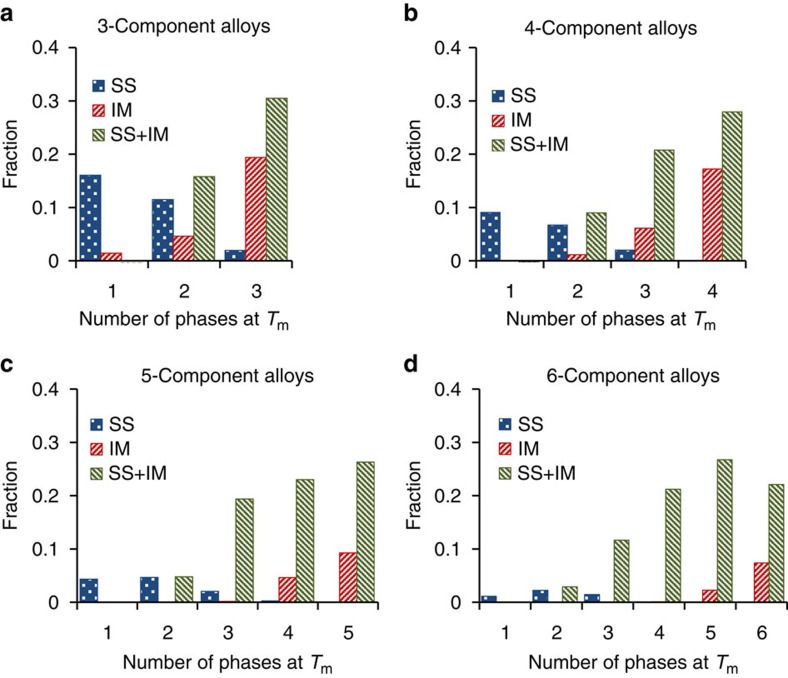
Distributions of *N*-component equimolar alloys by the number of phases at *T*_m_. Distributions of SS, IM and (SS+IM) alloys by the number of phases in (**a**) 3-, (**b**) 4-, (**c**) 5- and (**d**) 6- component alloy systems.

The amounts and fractions of SS, IM and (SS+IM) *N*-component equimolar alloys having different number of phases at *T*_m_ are shown in Table 1 and Fig. 2, respectively. Almost all single-phase alloys are SS alloys. In fact, single-phase IM alloys are only observed within 3-component (12 alloys) and 4-component (2 alloys) alloy categories. The fraction of single-phase SS alloys decreases with an increase in the number of alloying elements, from 16% in 3-component to 1.2% in 6-component alloys (Fig. 2). Among 3- to 5-component alloys, the number and fraction of SS alloys at *T*_m_ decrease rapidly with an increase in the number of phases. In 6-component alloy category, about half of SS alloys contain two phases. The maximum number of SS phases that coexist in a SS alloy is 4, and is found in very few 4-, 5- and 6-component alloys (Table 1). The amount and fraction of IM alloys and (SS+IM) alloys increase with increasing number of phases at *T*_m_ (see Table 1 and Fig. 2). The 5-component alloys containing 5 phases and 6-component alloys containing 5 or 6 phases contain only IM phases or IM+SS phases.

**Figure 3:**
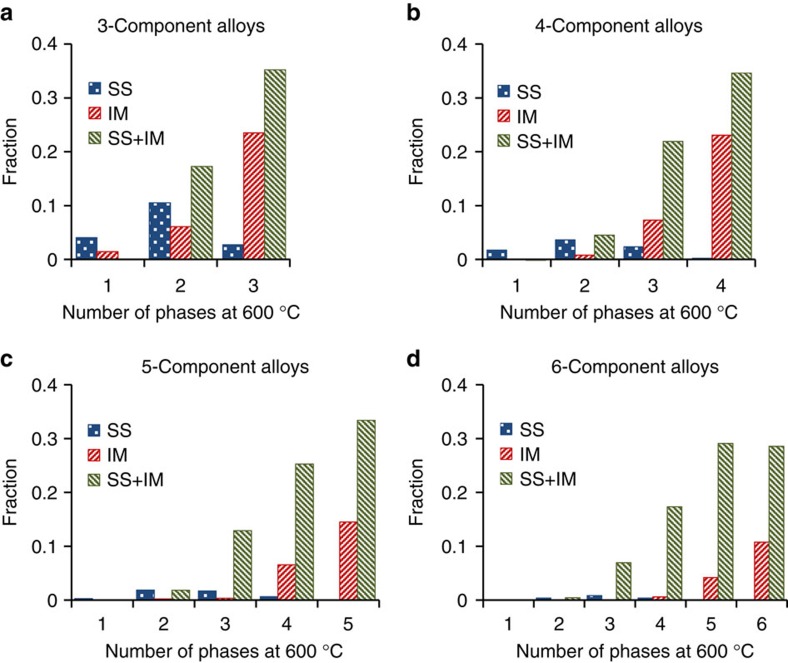
Distributions of *N*-component equimolar alloys by the number of phases at 600 °C. Distributions of SS, IM and (SS+IM) alloys by the number of phases in (**a**) 3-, (**b**) 4-, (**c**) 5- and (**d**) 6- component alloy systems.

The number and type of phases at 600 °C for *N*-component equimolar alloys are shown in Fig. 3. The fraction of single-phase SS alloys decreases considerably relative to *T*_m_. The fraction of single-phase IM alloys slightly increases in 3-component alloys and decreases to zero in more complex alloys. No single-phase 6-component alloys are present at 600 °C. With an increase in the number of phases, the fraction of SS alloys approaches a shallow maximum value at two or three phases, while the fractions of IM and (SS+IM) alloys drastically increase.

These results show that the probability of producing an equimolar alloy containing only SS phases decreases with an increase in the number of phases formed. Most of the SS alloys are single-phase, two-phase or three-phase alloys, while the majority of IM and SS+IM alloys contain three or more phases.

### The most common phases in equimolar alloys

**Figure 4:**
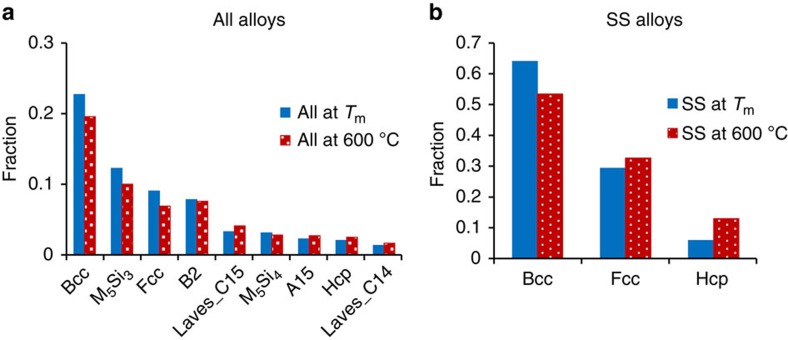
Most frequent phases at *T*_m_ and 600 °C. Fractions of different phases in (**a**) all alloys and (**b**) solid solution alloys.

About 372 different phases were identified in the processed equimolar alloys. The most frequent phases are BCC, FCC and HCP disordered SS phases and IM phases including B2, two silicide phases, M_5_Si_3_ and M_5_Si_4_, two Laves phases and A15 (Fig. 4a). The fractions of BCC, FCC, silicide and B2 phases are slightly higher and the fractions of other phases are slightly lower at *T*_m_ than at 600 °C. The phase distribution in SS alloys is shown in Fig. 4b. In these alloys, about 65% of all the phases present at *T*_m_ have BCC crystal structure, 29% have FCC and 6% have HCP crystal structure. The SS alloys at 600 °C have 53% of BCC phase, 32% of FCC phase and 15% of HCP phase.

### Design of equimolar alloys via *T*_m_ and *T*_use_

**Figure 5:**
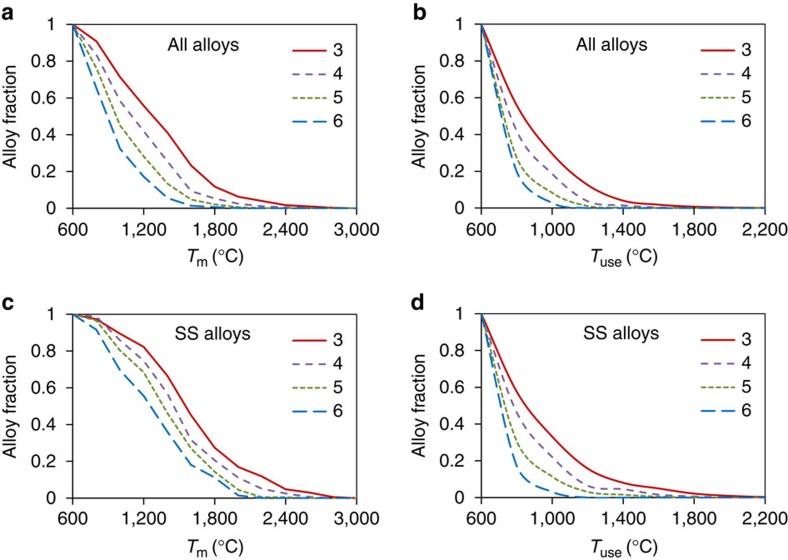
Cumulative distributions of *N*-component equimolar alloys by *T*_m_ and *T*_use_. (**a**) *T*_m_, all alloys, (**b**) *T*_use_, all alloys, (**c**) *T*_m_, SS alloys, (**d**) *T*_use_, SS alloys.

The maximum use temperature, *T*_use_, is a key consideration in alloy development. *T*_use_ depends on microstructure, but can be broadly estimated from the phase diagram. Here we take *T*_use_ to be the lowest of 0.8*T*_m_, and the temperature of the last first-order phase reaction below *T*_m_ (see Methods). Cumulative distributions of different category equimolar alloys by *T*_m_ and *T*_use_ are shown in Fig. 5a and Fig. 5b, respectively. The fraction of alloys rapidly decreases with an increase in *T*_m_ and *T*_use_ and the rate of the decrease is more rapid for alloys with a higher number of alloying elements. For example, the fractions of 3-, 4-, 5- and 6-component alloys with *T*_m_>1,200 °C are 56%, 42%, 28% and 17%, respectively, and those with *T*_m_>1,800 °C are 12%, 5%, 2% and 0.6%, respectively (Fig. 5a). The maximum *T*_m_ values for the 3-, 4-, 5- and 6-component alloys are 2,925 °C, 2,754 °C, 2,435 °C and 2,056 °C, respectively.

About 30, 19, 8 and 3% of the 3-, 4-, 5- and 6-component alloys have *T*_use_ >1,000 °C and only 2, 0.4, 0.1 and 0% of this category alloys have *T*_use_ >1,600 °C (Fig. 5b). The maximum *T*_use_ values registered for the 3-, 4-, 5- and 6-component alloys are 2,285 °C (MoTaW), 1,846 °C (MoNbTaTi), 1,677 °C (MoNbTaTiV) and 1,159 °C (MoNbReSiTiZr), respectively. These results show that both the solidus temperature and the maximum *T*_use_ noticeably decrease with an increase in the number of alloying elements.

The SS equimolar alloys show behaviour similar to that described above, however, the effect of the number of alloying elements on the cumulative distributions of alloys by *T*_m_ and *T*_use_ is even stronger, and a larger fraction of the SS alloys has higher *T*_m_ and *T*_use_ values (in comparison with all alloys; Fig. 5c,d). For example, about 33%, 23%, 12% and 4% of, respectively, 3-, 4-, 5- and 6-component SS alloys have *T*_use_ >1,000 °C. In total, 209 SS alloys have *T*_use_≥1,000 °C, among which 108 are 3-component alloys, 73 are 4-component alloys, 25 are 5-component alloys and only 3 are 6-component alloys.

### Design of equimolar alloys to give targeted properties

**Figure 6:**
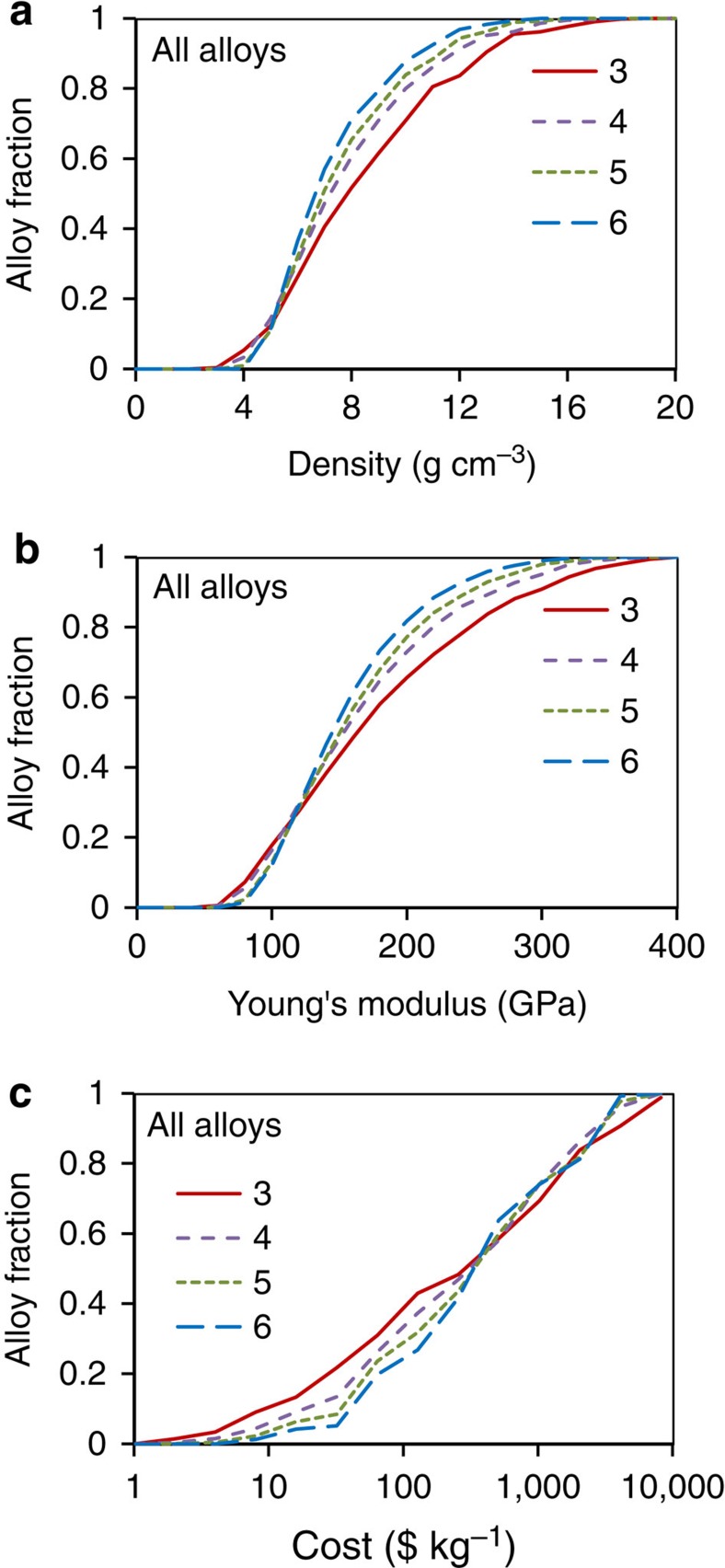
Cumulative distributions of the *N*-component equimolar alloys by their properties. (**a**) Density, (**b**) Young’s modulus and (**c**) cost.

The cumulative distributions of equimolar alloys by density, Young’s modulus and cost are given in Fig. 6a–c, respectively. Alloy densities vary from 2.37 to 18.9 g cm^−3^. From 71% (3-component alloys) to 88% (6-component alloys) of the alloys have densities <10 g cm^−3^ and from 30% (3-component alloys) to 36% (6-component alloys) have densities <6 g cm^−3^ (Fig. 6a). The Young’s modulus values of 3-, 4-, 5- and 6-component alloys are in the range of 54 to 400 GPa, 57 to 375 GPa, 65 to 348 GPa and 67 to 328 GPa, respectively (Fig. 6b). About 85% of all the alloys have Young’s moduli >100 GPa, and 18–34% (the percentage decreases with an increase in the number of alloying elements) have Young’s moduli >200 GPa. The alloy cost can be as low as $1 per kg and as high as $13,000 per kg. The fraction of ‘low-cost’ (<$100 per kg) alloys decreases with an increase in the number of alloying elements (Fig. 6c). For example, about 40% of 3-component and 26% of 6-component alloys cost <$100 per kg, and about 10% of 3-component and 2.4% of 6-component alloys cost <$10 per kg. The 3-component alloys have the maximum spread in properties for all three cumulative distributions, so that the highest and lowest values are obtained for ternary alloys.

The wide spread of physical and thermodynamic properties shown here provide an opportunity to identify compositions that meet particular application requirements. As an example, we identify alloys that meet the following set of properties: (a) no IM phases at *T*_m_; (b) *T*_use_ is ≥1,000 °C; (c) density is ≤10 g cm^−3^; (d) Young’s modulus is ≥100 GPa; and (e) cost is ≤$200 per kg. The alloys that meet these criteria are given in Tables 2 and 3. Among these, 42 alloys are single-phase BCC structures, 5 alloys are single-phase FCC structures, 2 alloys have 2 BCC phases and 2 alloys have BCC+HCP phases at *T*_m_. These alloys include 22 3-component, 23 4-component, 5 5-component and 1 6-component alloys. The present example suggests that increasing the number of alloying elements reduces the probability of meeting particular application requirements. Nevertheless, the present work identifies 51 equimolar alloy systems with *N* of 3–6 that show potential to satisfy aggressive goals for high-temperature structural metal alloys.

## Discussion

A major new finding of the present work is that the likelihood of forming equimolar alloys that contain only disordered SS phases decreases with increasing number of constituents, *N*. Both the fraction of *N*-component alloys that are SS and the total number of SS alloys decrease with increasing *N* (see Fig. 1a and Table 1). The same trend is seen at *T*_m_ and 600 °C. This result contradicts the primary concept of HEAs—that increasing *N* improves the stability of disordered SSs by increasing the configurational entropy of the alloy^1^,^2^. While SS alloys are found in some HEAs, many others frequently form IM phases^9^,^10^. With the number of reported HEAs now exceeding 100 (ref. 5), it becomes more evident that configurational entropy alone is not a sufficient criterion to govern the formation of SS phases in HEAs^11^,^12^,^13^,^14^. Phenomenological criteria, such as atomic radius difference, electronegativity difference, valence electron concentration and enthalpy of mixing have been introduced to explain these contradicting results^15^,^16^,^17^,^18^. These approaches have some success, and show observed trends in the types of phases formed, but they lack the accuracy and rigour of a fundamental thermodynamic criterion to predict phase selection in complex alloys.

The Gibbs free energy, *G*=*H*−*TS*, is the established thermodynamic criterion for phase selection, where *H* is enthalpy, *S* is entropy and *T* is absolute temperature. The Gibbs free energies for SS phases are described by enthalpies and entropies of mixing (*H*_mix_ and *S*_mix_), and IM phases are characterized by enthalpies and entropies of formation (*H*_f_ and *S*_f_). The Gibbs free energies for all competing phases must be estimated, and the phase (or phases) with the lowest *G* gives the stable microstructure for that alloy. *S*_mix_, also called configurational entropy, is easy to model^19^and *H*_mix_ has been estimated for wide range of atom pairs^20^. *S*_f_ for IM compounds is generally very small^21^, and is often estimated to be zero^8^. *H*_f_ of IM compounds is an essential term^8^,^11^ but is difficult to estimate—there are no general models. *H*_mix_ is sometimes used as a proxy for *H*_f_, but the differences between these values can be very large, and at best this approach gives only a relative ranking that cannot be used to determine *G*. Fundamental approaches to calculate *H*_f_ are available^22^ but are difficult to apply to over 100,000 alloys as in the present case. To overcome these barriers, here we use the CALPHAD approach to perform a quantitative comparison of all thermodynamic quantities (including formation enthalpies) needed to determine the stable phases for a given alloy.

CALPHAD predictions are reliable over composition ranges for which the databases have been built. In the present work, we often use the databases outside these composition ranges, and so the credibility of CALPHAD calculations for complex, equimolar alloys must be considered. Some comparisons have been made between CALPHAD predictions and experimentally observed phases of complex, equimolar alloys^14^,^23^,^24^,^25^,^26^. The agreement is good for the types of phases present. More detailed predictions (phase composition, volume fraction and transformation temperatures) show modest agreement, but this information is not used in the present work to improve credibility. As a final effort to improve credibility, here we only use results for calculations, where all bounding binary phase diagrams in the *N*-component system have complete and explicit descriptions in the database used for that calculation (see Methods). This stringent filter eliminates all but the 5,540 most credible calculations (representing 4,983 unique alloys) from the full field of 134,547 calculations (see Supplementary Table 2). All of our present results are based on these 5,540 most credible calculations.

The results of our CALPHAD calculations are compared with the phase compositions for 40 equimolar alloys so far reported in open literature (see Supplementary Table 3). Since the experimental observations are often made on as-cast product and the calculations represent thermodynamic equilibrium, some differences are expected. Supporting this, different studies sometimes measure different phases in the same alloy (for example, see entries for AlCoCrFeNi in Supplementary Table 3). Further, it may be difficult to distinguish experimentally between microstructures with a single BCC phase and two or more BCC phases if the lattice constants are similar. The same is true for other disordered structures (FCC and HCP), and for distinguishing between ordered and disordered phases based on the same crystal structure (for example, BCC and B2, FCC and L1_2_). Thus, although the first row in the table gives different results for AlCoCrCuFeNi between experimental (BCC+FCC) and calculated (FCC+B2+FCC) results, this is considered nominal agreement within the uncertainties in this comparison. Many of the rows show direct agreement, many show nominal agreement, and a smaller number show disagreement between observed and calculated phases. Overall the agreement is good. Based on these considerations, the present approach is judged to be sufficient to support the conclusions given here. Additional credibility checks and experimental validations are currently underway.

This major finding that the fraction of SS alloys decreases with increasing number of constituents is understood by recognizing that configurational entropy rises slowly with *N* (as ln(*N*)), while the number of binary interactions between element pairs increases much faster (as (*N*/2)(*N*−1)). For example, increasing *N* from 3 to 6 increases configurational entropy by 163%, while the number of binary interactions increases by 500%. This gap widens as *N* increases further. Thus, the probability that an *N*-component alloy has at least one element pair with a formation enthalpy that is sufficiently large to overcome the configurational entropy term increases more rapidly with *N* than the configurational entropy. Supporting this interpretation, we estimate *G* for a typical 6-component equimolar alloy at 900 K as −31.1 kJ mol^−1^ (see Methods). The median Gibbs energy for IM compounds is more negative (−36 kJ mol^−1^)^8^, and for some compounds like aluminides or silicides can be much smaller (down to −70 kJ mol^−1^), emphasizing the likelihood of forming an IM compound as *N* increases. This analysis is consistent with the Gibbs energy criterion that requires account of both enthalpy and entropy in phase selection.

A second finding of the present work is that extreme properties become more difficult to achieve in equimolar alloys as *N* increases. Thus the highest and lowest densities, Young’s moduli and cost are achieved in equimolar ternary alloys, and alloys with *N*=6 have tighter spreads. This is certainly expected for properties that can be estimated from a rule-of-mixtures for the atoms in the alloy such as density, modulus and cost. We find the same trend for *T*_m_ and *T*_use_ in the present work. However, this is not expected to be a general result, as *T*_m_ is not a linear function of composition. Local compositions can have *T*_m_ values that vary from a linear average of the bounding elements by ±50% or more. The present work considers only one composition in each alloy system—the equimolar alloy—and more extensive computations that include non-equimolar alloys are expected to uncover local compositions with much higher (and much lower) *T*_m_ and *T*_use_ than those indicated here. Exploration of non-equimolar alloys is a topic of future work. Although extreme values are more difficult to achieve in high-*N* alloys, a larger fraction of equimolar alloys with *N*=6 have densities in the useful range of 6 to 10 g cm^−3^ than do alloys with *N*=3, and a larger fraction with *N*=6 have attractive Youngs moduli in the range of 150 to 250 GPa. From this discussion, we expect that properties such as density, modulus and cost may be designed using intuitive, linear concepts, but that designing *T*_use_ is likely to require a more computationally intensive effort to explore non-equimolar composition space.

The present work shows that increasing *N* reduces the number of equimolar alloys that satisfy a selected set of design requirements. Although only 1 computed 6-component alloy meets the requirements chosen here, we nevertheless report 50 new alloy systems with 3≤*N*≤5 that satisfy the selection criteria and are recommended for further study. This dramatically expands the list of potential alloy systems for high-temperature structural applications, supporting the value of the present high-throughput calculations for accelerated alloy exploration. The present work is for equimolar alloys only, and extending to non-equimolar alloys is expected to significantly expand the list of candidate alloy systems. Further, we discard >95% of the calculated alloy systems to meet the highest standard for our credibility criterion (*f*_AB_=1). More extensive experimental validations may show that adequate agreement may be achieved with a less stringent credibility criterion (*f*_AB_<1), increasing the number of alloys for consideration. Finally, we observe that some alloys that satisfy our selection criteria have more than one phase and may include IM phases below *T*_use_. This is consistent with a rich experience in physical metallurgy, where most structural metals achieve a difficult balance of competing properties by careful control of multi-phase microstructures. Thus, a rich field of study of multi-phase alloys is suggested here, where producing single-phase alloys is not a major objective. This shifts the focus to developing logical approaches to interrogate phase diagrams as candidates for structural metals. As a vital companion to high-throughput calculations, the present work also calls for the development of high-throughput experimental techniques that are capable of dealing with the intrinsic length scales introduced by microstructure in candidate structural metal alloys.

In conclusion, the computational screening and analysis of thousands of equimolar alloy compositions for potential high-temperature structural applications have allowed us to identify new composition-dependent trends. The fraction of SS alloys noticeably decreases with an increase in the number of alloying elements, which can be understood by the competition between configurational entropy (favouring SS) and formation enthalpies (favouring IM phases). Almost all single-phase equimolar alloys are SS alloys, while the majority of the multi-phase alloys are (SS+IM) alloys. An increase in the number of alloying elements in equimolar alloys also reduces the available range of properties, such as the maximum *T*_use_, density, elastic modulus and cost. We identify 51 new equimolar alloy systems with 3–6 base elements as high-temperature structural metal candidates for additional study.

## Methods

### Combinations without repetition

The number of combinations (where the order of the objects doesn’t matter) of *R* objects chosen only once (repetition is not allowed) from a palette of *N* objects is


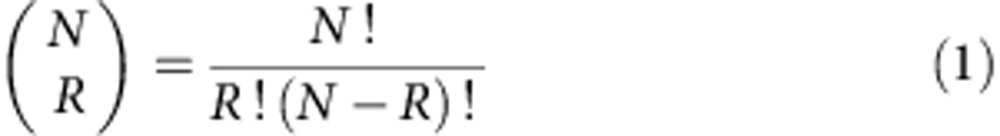


### Alloy screening via CALPHAD

A concept for accelerated assessment of MPEAs has recently been proposed^8^. Here we develop and apply this idea to quickly identify, screen and analyze structural alloys for use at moderate and high temperatures. We select a palette of 26 metallic elements that are most likely to produce the moderate and high-temperature properties ranges^8^ in MPEAs. The palette of elements is shown in Supplementary Table 1.

Phase diagrams are the road maps for materials design, and give essential information on the phases present at a given alloy chemistry and temperature. Most binary and some ternary phase diagrams have been determined experimentally, but multi-component systems remain largely unexplored. Experimental measurement of multi-component phase diagrams is impractical due to the tremendous amount of work involved. In recent years, integration of the CALPHAD approach with key experiments has been demonstrated as an effective approach to determine complicated multi-component phase diagrams^22^. The essence of the CALPHAD approach is to use experimental data from constituent binary and ternary phase diagrams to develop a thermodynamic database. This database is then used to calculate phase stability of multi-component systems. Thermodynamic parameters for quaternary and higher order systems are usually not considered in the construction of multi-component databases because interactions in higher order subsystems become negligibly weak^27^,^28^. A reliable account of higher order systems is obtained via extrapolation^29^.

We calculate phase diagrams using Pandat v.8.1 software and eight thermodynamic databases developed by CompuTherm LLC. The elements in each database that are also in our palette of elements are listed in Supplementary Table 1. Each database has a different subset of the 26 elements. We calculate a phase diagram for every equimolar alloy with three to six elements drawn from the databases in Supplementary Table 1. The number of 3-, 4-, 5- and 6-component alloys thus calculated are 4,746, 14,868, 37,725, and 79,661, respectively, giving a total of 134,547 phase diagram calculations (see Supplementary Table 2). The same alloy compositions can appear in different databases. For example, AlCrFe is present in all eight databases and AlCrFeMoNb is present in six databases (see Supplementary Table 1). Therefore, the number of unique alloys is smaller than the total number of the processed alloys, but still is a large number (see Supplementary Table 2). To facilitate this large number of calculations, scripts were written to generate batch files for CALPHAD analysis; to run multiple batch files through the Pandat software; to export thermodynamic data into separate text files and to create summary files with all data from the phase diagrams needed for subsequent analysis. Phase diagram calculations were run between 600–3,600 °C (873–3,873 K) at an interval of 20–30 °C. The output from each phase diagram includes the liquidus (*T*_liq_) and solidus (also called *T*_m_) temperatures; the first reaction temperature below *T*_m_; the last reaction temperature below *T*_m_; the number and types of phases at *T*_m_; and the number and types of phases at the lowest temperature (600 °C).

Each phase is identified by its crystal structure, and each microstructure (taken from a specific alloy at a specific temperature) is characterized as consisting of disordered SS phases, IM phases or a mixture of SS+IM. Many IM phases are stable over a range of compositions, indicating some ability to produce a SS, and some studies combine a select number of these ordered SS phases and SS phases in the same category^30^. However, ordered SS have two or more sublattices as does the parent IM phase and thus are clearly distinct from SS phases, which have only one lattice. In the present work, all ordered SS phases are considered as IM phases.

The data extracted from each phase diagram were evaluated against specific criteria to identify promising structural metal alloys. As fundamental requirements, *T*_m_ must be above the maximum *T*_use_, and no first-order phase transformations should occur below *T*_use_ to ensure the properties and part dimensions are stable during use. Based on this information, the maximum use temperature, *T*_use_, is calculated to be the lowest of 80% of the absolute *T*_m_ and the last first-order phase transformation temperature. As good ductility and toughness are often associated with a SS primary phase, we identified SS alloys, which do not contain IM phases, at least at *T*_m_.

Finally, we characterize SS alloys by the practical properties of density (*ρ*), modulus (*E*), cost (*P*) and *T*_use_. These properties are calculated using the rule of mixtures and the respective properties of pure elements


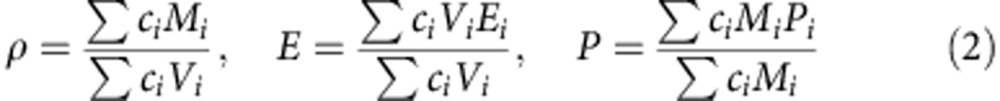


Here, *c*_*i*_, *M*_*i*_, *V*_*i*_, *E*_*i*_ and *P*_*i*_ are the atomic fraction, molar mass, molar volume, Young’s modulus and cost-per-kg of element *i*.

### Credibility criteria for CALPHAD calculations

CALPHAD calculations are well-established and are capable of producing accurate and reliable phase diagram information. These calculations rely on thermodynamic databases tailored for a specific alloy family, and the best results are obtained within the composition range where significant data have been used to build that database. Calculations may be performed with a given database outside of this preferred composition range, but the reliability of the calculations may be reduced. For example, the Fe database PanFe2013 includes the elements Fe, Al, Co, Cr and Ni. Calculated phase equilibria in the Co-Cr-Fe-Ni quaternary system may agree well with the experimental data, especially in the Fe corner, since the database has complete thermodynamic description for the Co-Cr, Co-Fe, Co-Ni, Cr-Fe, Cr-Ni and Fe-Ni binary systems and Co-Cr-Fe, Co-Fe-Ni and Cr-Fe-Ni ternary systems. This same database could be used to calculate phase diagrams of the Al-Co-Cr-Fe system, but the reliability of the calculations may be reduced because thermodynamic description of the Al-Co, Al-Cr and Al-Fe binary systems is not complete in the PanFe2013 database.

The current calculations are for equimolar alloys that are, by definition, in the centre of the phase diagram and may use thermodynamic databases in composition regimes where relatively little supporting thermodynamic data are available. To address this, we develop credibility criteria to indicate the confidence that may be placed in any given phase diagram calculation. We define credibility using two parameters: the fraction of fully thermodynamically assessed binary systems (*f*_AB_) and the fraction of fully assessed ternary systems (*f*_AT_) included in the database for the specific alloy under consideration. Consider the 5-component database ABCDE, where the A–B, A–C and B–C binary phase diagrams and the A–B–C ternary phase diagram have complete thermodynamic description in this database. In an *N*-component alloy, there are (*N*/2)(*N*−1) binary phase diagrams and (*N*/6)(*N*−1)(*N*−2) ternary phase diagrams (see Supplementary Table 4), so that 3 of 10 binaries and 1 of 10 ternaries are used to build the ABCDE database. For alloy ABC, all relevant binary and ternary diagrams are included in the ABCDE database, so that *f*_AB_=1 and *f*_AT_=1, and the credibility is high. However, the calculated diagram for the BCD alloy using the same database has *f*_AB_=1/3 and *f*_AT_=0. The phase diagram for alloy CDE has even lower credibility, where *f*_AB_=0 and *f*_AT_=0. Thus, phase diagrams calculated for different alloys using the same database can have different credibilities, and phase diagrams for the same alloy in different databases may also have different credibilities. For reference, the number and fraction of binary and ternary phase diagrams used to construct the thermodynamic databases used in the present study are shown in Supplementary Table 5. The list of binary and ternary systems assessed in the thermodynamic databases used here can be found at www.computherm.com.

CALPHAD calculations for an *N*-component alloy are considered to be fully credible if the thermodynamic data are available for all the binary and ternary systems embedded in this alloy. Supplementary Figure 1 shows that *f*_AB_=1 for a number of alloys considered here, but *f*_AT_=1 only for a few alloys with *N*=3. For the alloys with *N*>3, *f*_AT_ is always <1. Thus, in the present work, we consider analysis for alloys with *f*_AB_=1 to be the most credible among all the analyzed alloys and only the alloys with *f*_AB_=1 and any values of *f*_AT_ are evaluated by other criteria in this paper. Alloys with *f*_AB_<1 are excluded from further analysis.

### Estimate of *G* for a 6-component equimolar alloy

We estimate *G* for a 6-component equimolar alloy using *G*=*H*_mix_−*TS*_mix_. *S*_mix_ is approximated as 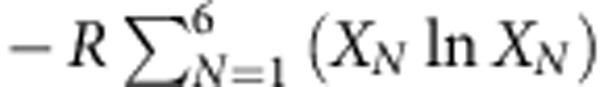 for an ideal solution, where *X*_N_=1/6. We use *T*=900 K to represent a temperature at which kinetic processes become sufficiently sluggish to practically block further phase transformations. To approximate the enthalpy of mixing, we use the average value of *H*_mix_=−17.7 kJ mol^−1^ from ref. 20. The final value from this estimate is *G*=−31.1 kJ mol^−1^.

## Author contributions

All the authors conceived the project and participated in the discussion of the obtained results. In addition, O.N.S. conducted CALPHAD calculations and analyzed the data; O.N.S. and D.B.M. wrote the paper; J.D.M. and C.W. wrote the computer programmes to process the CALPHAD results, calculate other alloy properties and conduct the analysis of the calculated properties against the selected criteria.

## Additional information

**How to cite this article:** Senkov, O. N. *et al*. Accelerated exploration of multi-principal element alloys with solid solution phases. *Nat. Commun.* 6:6529 doi: 10.1038/ncomms7529 (2015).

## Supplementary Material

Supplementary Information Supplementary Figures 1, Supplementary Tables 1-5 and Supplementary References.

## Figures and Tables

**Table 1:** Number of different groups of unique equimolar alloys with *f*_AB_=1.

Number of components	3	4	5	6
Number of alloys	742	1,405	1,548	1,288
Number of SS alloys at *T*_m_	221	253	183	69
Single-phase SS alloys	119	127	69	16
Two-phase SS alloys	86	94	74	31
Three-phase SS alloys	16	30	34	20
Four-phase SS alloys	0	2	6	2
Number of SS alloys at 600 °C	129	114	75	24
Single-phase SS alloys	31	25	6	0
Two-phase SS alloys	78	52	30	6
Three-phase SS alloys	21	33	28	12
Four-phase SS alloys	0	4	11	6
Number of IM alloys at *T*_m_	191	347	226	130
Single-phase IM alloys	12	2	0	0
Two-phase IM alloys	35	17	2	0
Three-phase IM alloys	144	87	5	0
Four-phase IM alloys	0	241	74	3
Five-phase IM alloys	0	0	145	31
Six-phase IM alloys	0	0	0	96
Number of IM alloys at 600 °C	231	438	339	204
Single-phase IM alloys	11	0	0	0
Two-phase IM alloys	46	12	5	0
Three-phase IM alloys	174	103	7	1
Four-phase IM alloys	0	323	103	9
Five-phase IM alloys	0	0	224	55
Six-phase IM alloys	0	0	0	139

SS, solid solution.

**Table 2:** Selected ternary equimolar alloys that are SS at *T*_m_.

**Alloy ID**	***T***_**m**_ **(°C)**	***T***_**use**_ **(°C)**	**Phases at** ***T***_**m**_	**Phases at 600 °C**	***ρ*** **(g cm**^−3^**)**	***E*** **(GPa)**	***P*** **($ per kg)**
AlCrFe	1,399	1,065	BCC	BCC	5.54	173	4
AlCrMn	1,325	1,005	BCC	BCC	5.45	170	5
AlCrMo	1,656	1,009	BCC	BCC+AlMo_3_+Al_8_Mo_3_	6.57	218	63
AlCrTi	1,663	1,020	BCC	Laves_C14	4.55	142	8
AlCrV	1,660	1,273	BCC	BCC	5.08	148	90
AlFeV	1,585	1,213	BCC	BCC	5.26	128	84
AlMnV	1,491	1,138	BCC	BCC	5.18	125	86
AlTiW	1,618	1,173	BCC+BCC	BCC+TiAl	8.59	194	19
CoCrNi	1,445	1,101	FCC	FCC	8.28	231	17
CoMoNi	1,382	1,051	FCC	FCC+Mu	9.43	256	61
CrFeNi	1,329	1,009	FCC	FCC+BCC	7.96	231	8
CrFeV	1,547	1,183	BCC	BCC	7.01	202	73
CrMnV	1,551	1,186	BCC	BCC	6.89	198	75
CrMoNb	1,978	1,165	BCC	BCC+Laves_C15+BCC	8.78	227	115
CrMoTi	2,348	1,824	BCC	BCC	7.19	233	59
FeMnV	1,405	1,069	BCC	BCC	7.11	176	70
MoNbTi	2,242	1,739	BCC	BCC	7.67	177	117
MoNbV	2,272	1,763	BCC	BCC	8.40	185	160
MoTiV	1,977	1,527	BCC	BCC	6.87	190	114
MoVY	1,458	1,112	BCC+HCP	BCC+HCP	6.27	144	115
MoWY	1,486	1,134	BCC+HCP	BCC+HCP	9.52	213	55
NbTiV	1,815	1,397	BCC	BCC	6.44	115	148

SS, solid solution.

The alloys have *T*_use_≥1,000 °C, density *ρ*≤10 g cm^−3^, Young’s modulus *E*≥100 GPa and cost *P*≤$200 per kg.

**Table 3:** Selected four to six component equimolar alloys that are SS at *T*_m_.

**Alloy ID**	***T***_**m**_ **(°C)**	***T***_**use**_ **(°C)**	**Phases at** ***T***_**m**_	**Phases at 600 °C**	***ρ*** **(g cm^−3^)**	***E*** **(GPa)**	***P*** **($ per kg)**
AlCrFeMn	1,372	1,043	BCC	BCC	5.99	179	3
AlCrFeMo	1,482	1,131	BCC	BCC+A15+L1_2_	6.85	217	48
AlCrFeV	1,578	1,208	BCC	BCC	5.69	162	63
AlCrMnMo	1,405	1,069	BCC	BCC+AlMo_3_	6.77	214	49
AlCrMnTi	1,357	1,031	BCC	BCC	5.16	154	6
AlCrMnV	1,517	1,159	BCC	BCC	5.62	159	64
AlCrMoV	1,715	1,165	BCC	BCC+AlMo_3_	6.47	197	99
AlCrVW	1,749	1,345	BCC	BCC+BCC	8.96	219	51
AlFeMnV	1,467	1,119	BCC	BCC	5.76	144	60
AlFeMoV	1,673	1,252	BCC	BCC+AlMo_3_	6.60	182	95
AlFeTiV	1,470	1,122	BCC	BCC	5.04	125	65
AlMnTiV	1,497	1,143	BCC	BCC	4.98	123	66
AlMoNbV	2,051	1,586	BCC	BCC	6.92	155	144
AlNbVW	1,880	1,053	BCC	BCC+BCC	9.18	176	91
CoCrFeNi	1,422	1,083	FCC	FCC	8.18	226	13
CoFeMoNi	1,338	1,016	FCC	FCC+Mu	9.06	245	48
CrFeMnV	1,452	1,107	BCC	BCC	7.13	201	55
CrFeTiV	1,577	1,179	BCC	BCC+Laves_C14	6.21	175	59
CrMnTiV	1,406	1,070	BCC	BCC	6.14	172	60
CrMoNbV	1,931	1,174	BCC	BCC+BCC	8.16	204	133
CrMoTiV	1,927	1,487	BCC	BCC	6.94	208	92
FeMoTiV	1,409	1,020	BCC	BCC+Laves_C14+FeTi	7.07	194	89
MoNbTiV	2,071	1,602	BCC	BCC	7.34	167	136
AlCrFeMnV	1,485	1,133	BCC	BCC	6.02	168	49
AlCrFeMoV	1,660	1,123	BCC	BCC+AlMo3	6.70	199	79
AlCrFeMoW	1,512	1,090	BCC+BCC	BCC+BCC+L1_2_+A15	9.60	259	37
AlCrMnTiV	1,482	1,131	BCC	BCC	5.35	149	53
AlCrNbVW	1,732	1,331	BCC	BCC+BCC	8.87	192	81
AlCrMoNbVW	1,915	1,120	BCC	BCC+BCC	9.10	215	86

SS, solid solution.

The alloys have *T*_use_≥1,000 °C, density *ρ*≤10 g cm^−3^, Young’s modulus *E*≥100 GPa and cost *P*≤$200 per kg.
