# Supplementary material for: Accelerated exploration of multi-principal element alloys with solid solution phases
Source: Nat Commun. 2015 Mar 5;6:6529. doi: 10.1038/ncomms7529 (PMC4366518; doi:10.1038/ncomms7529)
Supplement: Supplementary Information — Supplementary Figures 1, Supplementary Tables 1-5 and Supplementary References. [file ncomms7529-s1.pdf]

SUPPLEMENTARY INFORMATION

This file contains Supplementary information to the manuscript titled “Accelerated Exploration of Multi-Principal Element Alloys with Solid Solution Phases,” by O.N. Senkov, J. D. Miller, D.B. Miracle and C. Woodward, Air Force Research Laboratory, Materials and Manufacturing Directorate, Wright-Patterson AFB, Ohio 45433, USA. The manuscript is published in Nature Communications in 2015

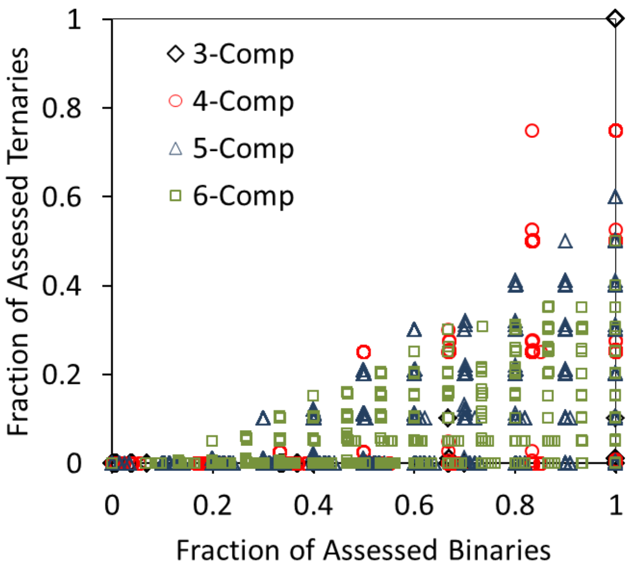

Supplementary Figure 1. Fractions of assessed binary and ternary systems for the studied N-component alloys.

Supplementary Table 1. The palette of selected elements and the elements in the thermodynamic databases.

**Database**	**#**	**Elements Included in Palette or Database**																									
**Palette of Elements**	**26**	Ag	Al	Co	Cr	Cu	Dy	Fe	Gd	Hf	Lu	Mg	Mn	Mo	Nb	Ni	Re	Rh	Ru	Sc	Si	Ta	Ti	V	W	Y	Zr
**PanAl2013**	**16**	Ag	Al		Cr	Cu		Fe	Gd	Hf		Mg	Mn			Ni				Sc	Si		Ti	V		Y	Zr
**PanCo2013**	**9**		Al	Co	Cr			Fe						Mo		Ni	Re					Ta			W		
**PanFe2013**	**15**		Al	Co	Cr	Cu		Fe				Mg	Mn	Mo	Nb	Ni					Si		Ti	V	W		Zr
**PanMo2013**	**10**		Al		Cr			Fe		Hf			Mn	Mo			Re				Si		Ti				Zr
**PanNb2013**	**11**		Al		Cr			Fe		Hf				Mo	Nb		Re				Si		Ti		W		Zr
**PanNi2013**	**17**		Al	Co	Cr	Cu		Fe		Hf			Mn	Mo	Nb	Ni	Re		Ru		Si	Ta	Ti		W		Zr
**PanTi2013**	**12**		Al		Cr	Cu		Fe						Mo	Nb	Ni					Si	Ta	Ti	V			Zr
**PanSol (MT)**	**19**	Ag	Al	Co	Cr	Cu	Dy	Fe			Lu	Mg	Mn	Mo	Nb	Ni				Sc	Si		Ti	V		Y	Zr
**PanSol (HT)**	**17**	Ag	Al	Co	Cr					Hf				Mo	Nb	Ni	Re	Rh	Ru		Si	Ta	Ti	V	W		Zr

Supplementary Table 2. Numbers of analyzed *N*-component equimolar alloys. Eight thermodynamic databases are used for the CALPHAD analysis and the numbers of 3 to 6 component alloys processed with the use of *n* databases, as well as the numbers of processed unique equimolar alloys, are shown.

Number of components, *N*	3	4	5	6
Total number of unique CALPHAD calculations	4746	14868	37725	79661
Number of the alloys common in *n* databases				
$n = 1$	1350	6588	24183	61431
$n = 2$	598	2039	4062	6401
$n = 3$	272	711	1080	1302
$n = 4$	162	308	384	307
$n = 5$	98	121	102	49
$n = 6$	14	20	15	7
$n = 7$	22	16	6	1
$n = 8$	1	0	0	0
Total number of unique alloys	2517	9803	29832	69498

Supplementary Table 3. Phases present in reported equimolar high entropy alloys. Comparison of experimental and calculated results.

Experimental Results				CALPHAD Calculations (Current Work)*	
**Alloy**	**Condition**	**Phases Reported**	**Ref.**	**Phases at T_m**	**Phases at 600°C**
AlCoCrCuFeNi	As-Cast	Bcc + Fcc	1	Fcc+B2+Fcc	B2+Fcc+Bcc+Bcc
AlCoCrCuNi	As-Cast	Bcc + Fcc	2	B2+Fcc+Fcc+Bcc	B2+Fcc+Sigma+Hcp
AlCoCrCuNi	As-Cast	Bcc + Fcc + (B2)	3	B2+Fcc+Fcc+Bcc	B2+Fcc+Sigma+Hcp
AlCoCrCuNiTi	As-Cast	Bcc + Cu + Cr?	4	Bcc+B2+B2+Fcc+Ni_3Ti	Bcc+B2+B2+Fcc+Ni_3Ti
AlCoCrFeNi	As-Cast	Bcc+B2	5,6	B2+Bcc+Fcc	B2+Sigma+Bcc
AlCoCrFeNi	As-Cast	B2 + $L1_2$	7	B2+Bcc+Fcc	B2+Sigma+Bcc
AlCoCrFeNiTi	As-Cast	Bcc + B2	6	Bcc+B2+B2+ C14+Fcc	Bcc+B2+B2+ C14+Ni_3Ti
AlCoCuNi	As-Cast	Bcc + Fcc	2	B2+Fcc	B2+Fcc+Fcc
AlCrCuFeMnNi	As-Cast	Bcc	8	Bcc+B2+Fcc+Fcc	Bcc+B2+Fcc+Fcc
AlCrCuFeNi	As-Cast	Fcc + Fcc	9	Bcc+B2+Fcc	B2+Fcc+Sigma
AlCrCuFeNiTi	As-Cast	Bcc + Fcc	10	Bcc+Fcc+C14+ B2+C15+NiTi	Bcc+Fcc+ C14+B2+ C15+NiTi
AlCrMnNbTi	As-Cast	Laves + unknown	11	Bcc	Bcc+ C15
AlCrMnNbV	As-Cast	Laves + unknown	11	Bcc	Bcc+ C14
AlCrMnTiV	As-Cast	B2	11	B2	B2
AlCrMoSiTi	As-Cast	B2 + Mo_5Si_3	12	Mo_5Si_3+Bcc	Mo_5Si_3+Bcc+Al_8Mo_3
AlCrNbTiV	As-Cast	B2	11	Bcc	Bcc+B2
AlCrTiVZr	As-Cast	Compounds	11	Bcc+B2	Bcc+ C15+AlZr+$D0_{19}$
AlMnNbTiV	As-Cast	B2 + Laves	11	Bcc	Bcc+$D0_{19}$+Laves_C14
AlTiVYZr	As-Cast	Compounds	13	AlY_2+Al_2Zr_3+Bcc+Hcp	AlY_2+Al_2Zr_3+Bcc+Hcp
CoCrCuFeMn	Annealed	Fcc + Fcc	14	Fcc+Bcc+Fcc	Fcc+Bcc+Fcc
CoCrCuFeMnNi	As-Cast	Fcc	15	Fcc+Fcc	Fcc+Fcc+Bcc
CoCrCuFeNi	As-Cast	Fcc	1,9	Fcc+Fcc	Fcc+Fcc+Bcc+Sigma
CoCrCuFeNiTi	As-cast	Fcc + Laves	16	Bcc + Fcc + C14+Ni_3Ti	Bcc+Fcc+ C14+Ni_3Ti+Bcc
CoCrFeMnNi	Annealed	Fcc	14	Fcc	Fcc+Bcc
CoCrFeMnNi	As-cast	Fcc	15	Fcc	Fcc+Bcc
CoCrFeNi	As-cast	Fcc	17	Fcc	Fcc+Sigma
CoCrMnNiV	Annealed	Fcc	14	Bcc+Fcc	Bcc+Fcc
CoCuFeNiV	As-Cast	Fcc	13	Fcc+Fcc	Fcc+Fcc
CoFeMnNiV	Annealed	Fcc	14	Fcc	Fcc
CoFeMnMoNi	Annealed	Fcc	14	Fcc	Fcc+Bcc+Mu
CrCuFeMnNi	As-Cast	Fcc + Bcc	9	Fcc+Bcc+Fcc	Fcc+Bcc+Fcc
CrCuFeMoNi	As-Cast	Fcc	9	Bcc+Fcc+Bcc	Bcc+Fcc
CrCuFeNiZr	As-Cast	Bcc	9	Bcc+Fe_2Zr+Fcc+$Ni_{10}Zr_7$	Bcc+Fe_2Zr+Fcc+$Ni_{10}Zr_7$+$Ni_{21}Zr_8$
CrFeMnNiTi	Annealed	α-Mn+IMs	14	Bcc+ C14+Ni_3Ti+NiTi	Bcc+C14+Ni_3Ti+ NiTi+Cr_3Mn_5
CrMnNbTiV	As-Cast	Laves + unknown	11	Bcc+ C14	Bcc+ C14
CrNbTiVZr	Annealed	Bcc+Laves	18	Bcc	Bcc+ C15+Bcc
CrNbTiZr	Annealed	Bcc+Laves	19	Bcc+ C15	Bcc+ C15

HfNbTaTiZr	Annealed	Bcc	[20]	Bcc	Bcc+Hcp
MoNbTaVW	Annealed	Bcc	[21]	Bcc	Bcc+Bcc
MoNbTaW	Annealed	Bcc	[21]	Bcc	Bcc
MoNbTiVZr	As-Cast	Bcc	[22]	Bcc	Bcc+Bcc
NbTiVZr	Annealed	Bcc	[18]	Bcc	Bcc+Bcc

*Strukturbericht symbols are used to identify Laves (C14 and C15), ClCs-type (B2), and $AuCu_3$-type ($L1_2$) IM phases.

Supplementary Table 4. Total number of binary and ternary systems in N-component alloys.

Number of components	3	4	5	6	*N*
Number of binary systems	3	6	10	15	(N/2)(N-1)
Number of ternary systems	1	4	10	20	(N/6)(N-1)(N-2)

Supplementary Table 5. Levels of assessment of the thermodynamic databases. The number of elements and the number and fraction of binary and ternary systems fully assessed in the thermodynamic databases used in the present work are shown.

**Database**	**Number of elements**	**Number of assessed binaries**	**Fraction of assessed binaries**	**Number of assessed ternaries**	**Fraction of assessed ternaries**
PanAl2013	16	21	0.18	15	0.027
PanCo2013	9	36	1.00	17	0.202
PanFe2013	15	15	0.14	10	0.020
PanMo2013	10	30	0.67	8	0.067
PanNb2013	11	46	0.84	12	0.073
PanNi2013	17	39	0.29	31	0.046
PanTi2013	12	40	0.61	1	0.005
PanSol2013	26	189	0.58	0	0.00

SUPPLEMENTARY REFERENCES

1 Yeh, J.-W. *et al.* Nanostructured high-entropy alloys with multiple principal elements: Novel alloy design concepts and outcomes. *Adv. Eng. Mat.* **6**, 299-303 (2004).

2 Yeh, J. W., Chang, S. Y., Hong, Y. D., Chen, S. K. & Lin, S. J. Anomalous decrease in X-ray diffraction intensities of Cu–Ni–Al–Co–Cr–Fe–Si alloy systems with multi-principal elements. *Materials Chemistry and Physics* **103**, 41–46 (2007).

3 Hsu, U. S. *et al.* Alloying behavior of iron, gold and silver in AlCoCrCuNi-based equimolar high-entropy alloys. *Mater. Sci. Eng. A* **460-461**, 403-408, doi:10.1016/j.msea.2007.01.122 (2007).

4 Hu, Z., Zhan, Y., Zhang, G., She, J. & Li, C. Effect of rare earth Y addition on the microstructure and mechanical properties of high entropy AlCoCrCuNiTi alloys. *Materials and Design* **31**, 1599-1602, doi:10.1016/j.matdes.2009.09.016 (2010).

5 Zhang, Y., Ma, S. G. & Qiao, J. W. Morphology Transition from Dendrites to Equiaxed Grains for AlCoCrFeNi High-Entropy Alloys by Copper Mold Casting and Bridgman Solidification. *Metall. Mater. Trans. A* **43A**, 2625-2630, doi:10.1007/s11661-011-0981-8 (2012).

6 Zhou, Y. J., Zhang, Y., Wang, Y. L. & Chen, G. L. Solid solution alloys of AlCoCrFeNi Tix with excellent room-temperature mechanical properties. *Applied Physics Letters* **90**, doi:10.1063/1.2734517 (2007).

7 Li, C., Zhao, M., Li, J. C. & Jiang, Q. B2 structure of high-entropy alloys with addition of Al. *Journal of Applied Physics* **104**, doi:10.1063/1.3032900 (2008).

8 Chen, H.-Y. *et al.* Effect of the substitution of Co by Mn in Al-Cr-Cu-Fe-Co-Ni high-entropy alloys. *Annales de Chimie: Science des Materiaux* **31**, 685-698, doi:10.3166/acsm.31.685-698 (2006).

9 Li, C., Li, J. C., Zhao, M. & Jiang, Q. Effect of alloying elements on microstructure and properties of multiprincipal elements high-entropy alloys. *J. Alloys Cmpds* **475**, 752-757 (2009).

10 Chen, M., Liu, Y., Li, Y. & Chen, X. Microstructure and mechanical properties of AlTiFeNiCuCrx high-entropy alloy with multi-principal elements. *Jinshu Xuebao/Acta Metallurgica Sinica* **43**, 1020-1024 (2007).

11 Cotton, J. M. & Kaufman. Unpublished work. (2014).

12 Yang, X. & Zhang, Y. Prediction of high-entropy stabilized solid-solution in multi-component alloys. *Mat. Chem. Phys.* **132**, 233-238 (2012).

13 Zhang, Y., Zhou, Y. J., Lin, J. P., Chen, G. L. & Liaw, P. K. Solid-Solution Phase Formation Rules forMulti-component Alloys. *Adv. Eng. Mat.* **10**, 534-538 (2008).

14 Otto, F., Yang, Y., Bei, H. & George, E. P. Relative effects of enthalpy and entropy on the phase stability of equiatomic high-entropy alloys. *Acta mater.* **61**, 2628-2638 (2013).

15 Cantor, B., Chang, I. T. H., Knight, P. & Vincent, A. J. B. Microstructural development in equiatomic multicomponent alloys. *Mat. Sci. Eng. A* **375-377**, 213-218 (2004).

16 Wang, X. F., Zhang, Y., Qiao, Y. & Chen, G. L. Novel microstructure and properties of multicomponent CoCrCuFeNiTix alloys. *Intermetallics* **15**, 357-362 (2007).

17 Hsu, Y.-J., Chiang, W.-C. & Wu, J.-K. Corrosion behavior of FeCoNiCrCux high-entropy alloys in 3.5% sodium chloride solution. *Mat. Chem. Phys.* **92**, 112-117 (2005).

18 Senkov, O. N., Senkova, S. V., Miracle, D. B. & Woodward, C. Mechanical properties of low-density, refractory multi-principal element alloys of the Cr–Nb–Ti–V–Zr system. *Mat. Sci. Eng. A* **565**, 51-62 (2013).

19 Senkov, O. N., Senkova, S. V., Woodward, C. & Miracle, D. B. Low-density, refractory multi-principal element alloys of the Cr–Nb–Ti–V–Zr system: Microstructure and phase analysis. *Acta mater.* **61**, 1545-1557 (2013).
20 Senkov, O. N., Scott, J. M., Senkova, S. V., Miracle, D. B. & Woodward, C. F. Microstructure and room temperature properties of a high-entropy TaNbHfZrTi alloy. *J. Alloys Cmpds* **509**, 6043-6048 (2011).
21 Senkov, O. N., Wilks, G. B., Miracle, D. B., Chuang, C. P. & Liaw, P. K. Refractory high-entropy alloys. *Intermetallics* **18**, 1758-1765 (2010).
22 Zhang, Y., Yang, X. & Liaw, P. K. Alloy design and properties optimization of high-entropy alloys. *JOM* **64**, 830-838, doi:10.1007/s11837-012-0366-5 (2012).
